# Cellular collusion: cracking the code of immunosuppression and chemo resistance in PDAC

**DOI:** 10.3389/fimmu.2024.1341079

**Published:** 2024-05-16

**Authors:** Chiara Musiu, Francesca Lupo, Antonio Agostini, Gabriella Lionetto, Michele Bevere, Salvatore Paiella, Carmine Carbone, Vincenzo Corbo, Stefano Ugel, Francesco De Sanctis

**Affiliations:** ^1^ Department of Medicine, University of Verona, Verona, Italy; ^2^ Department of Engineering for Innovation Medicine, University of Verona, Verona, Italy; ^3^ Medical Oncology, Department of Translational Medicine, Catholic University of the Sacred Heart, Rome, Italy; ^4^ Medical Oncology, Department of Medical and Surgical Sciences, Fondazione Policlinico Universitario Agostino Gemelli Istituti di Ricovero e Cura a Carattere Scientifico (IRCCS), Rome, Italy; ^5^ General and Pancreatic Surgery Unit, Pancreas Institute, University of Verona, Verona, Italy; ^6^ ARC-Net Research Centre, University of Verona, Verona, Italy

**Keywords:** PDAC - pancreatic ductal adenocarcinoma, immunotherapy, TME (tumor microenvironment), cancer associated fibroblast (CAF), MDSC (myeloid-derived suppressor cells), TILs (tumor infiltrating lymphocytes), immunosuppression

## Abstract

Despite the efforts, pancreatic ductal adenocarcinoma (PDAC) is still highly lethal. Therapeutic challenges reside in late diagnosis and establishment of peculiar tumor microenvironment (TME) supporting tumor outgrowth. This stromal landscape is highly heterogeneous between patients and even in the same patient. The organization of functional sub-TME with different cellular compositions provides evolutive advantages and sustains therapeutic resistance. Tumor progressively establishes a TME that can suit its own needs, including proliferation, stemness and invasion. Cancer-associated fibroblasts and immune cells, the main non-neoplastic cellular TME components, follow soluble factors-mediated neoplastic instructions and synergize to promote chemoresistance and immune surveillance destruction. Unveiling heterotypic stromal-neoplastic interactions is thus pivotal to breaking this synergism and promoting the reprogramming of the TME toward an anti-tumor milieu, improving thus the efficacy of conventional and immune-based therapies. We underscore recent advances in the characterization of immune and fibroblast stromal components supporting or dampening pancreatic cancer progression, as well as novel multi-omic technologies improving the current knowledge of PDAC biology. Finally, we put into context how the clinic will translate the acquired knowledge to design new-generation clinical trials with the final aim of improving the outcome of PDAC patients.

## Introduction

Pancreatic ductal adenocarcinoma (PDAC), with an 11% 5-year overall survival, represents a great clinical challenge (1). Increased insurgency related to improved life expectancy at birth and metabolic co-morbidities, as well as limited surgical chances, frequent relapses and high tumor resilience represent peculiar features that underscore an unmet clinical need and project this disease toward the second leading cancer-dependent death cause, in the USA, by 2040 (2). High lethality is mainly attributed to late diagnosis. Most of the patients face no symptoms until an advanced or metastatic evolutionary disease stage, whereas the pancreas anatomical position does not allow for routine screening test; in addition, germline mutations and known risk factors (e.g. pancreatitis) contribute to a dismal amount of PDAC. Late diagnosis restricts surgery to only 10-20% of patients; nonetheless almost 80% of them will relapse within 2 years underscoring that micro metastases are already present at resection (3).

Tumor microenvironment (TME) represents a critical tumor component, composed of normal cells of different ontogeny and function which can support or dampen tumor proliferation, stemness and invasion as well as sustain primary and acquired resistance to therapy (4). This complex landscape includes immune cells (from both innate and adaptive arms), fibroblasts, endothelial cells, dense extracellular matrix (ECM)and additional factors (e.g. cytokines, growth factors, extracellular vesicles) which establish multiple heterotypic interactions defining and leveraging neoplastic phenotypes. Tumor cells play a pivotal role in orchestrating TME by secreting a multitude of soluble factors altering physiological processes to a pathological state, including hematopoiesis (5), inflammation, angiogenesis (6) and hijacking the recruitment and polarization of specific stromal subsets (7) with the final aim to support cancer outgrowth. Notably, TME composition and function are highly heterogeneous between patients with similar histopathological characteristics and even within the same patient (8). Indeed, histological and molecular analysis unveil the coexistence of multiple “subTMEs” which are linked to tumor differentiation, immune infiltrate, response to treatment and clinical outcome (9). These subTMEs can be categorized as “reactive”, when infiltrated by heterogenous cancer-associated fibroblast and immune cell subsets and associated with more aggressive basal epithelial phenotype; or matrix-rich “deserted”, in which the infiltration of activated CAF and immune cells is less prominent, and this phenotype is usually associated with resistance to chemotherapy. Notably, the coexistence of multiple and heterogenous subTMEs is not casual and provides malignant tumor advantages (9).

Fibroblasts represent a peculiar component of PDAC TME. They can be activated to CAFs by a plethora of stimuli, including extracellular matrix composition and stiffness, DNA damage, cytokines such as TGFβ, IL6, IL1β and tyrosine kinase receptors such as PDGF and FGF receptors (10). CAFs can support tumor progression both directly, by matrix remodeling or tumor metabolic reprogramming and indirectly by acting on other stromal components by restraining immune responses and promoting angiogenesis (10). CAF targeting represents a clinical challenge because of their heterogeneity in both composition and pro-tumor/anti-tumor functions. CAFs are indeed categorized according to phenotypic markers and function: the ones exhibiting a matrix-producing contractile matrix producing function (myCAFs) shares a myofibroblast phenotype; fibroblasts with immunomodulatory ability, named inflammatory CAFs (iCAFs), express inflammatory cytokines, such as IL6. my-CAFs are induced by TGFβ and are phenotypically characterized by elevated α-SMA expression. Fibroblast activation protein α (FAP)^+^-CAFs and antigen-presenting CAFs (apCAFs) are both endowed with immunomodulatory properties. Notably, most of the CAF subsets are shared in both pancreatic cancer and other solid tumors associated with metastasis and worse prognosis (11), strengthening the relationship of peculiar TME components in supporting invasive abilities and spreading.

From an immunological point of view pancreatic cancer is moderately antigenic (12) and poorly immunogenic (13), a hallmark of cancer progression (14). Indeed, neoplastic cells abrogate cancer immune surveillance by both dampening the generation and maturation of functional dendritic cells (15) and promoting differentiation of both monocytes and granulocytes toward the generation of immune-suppressive myeloid-derived suppressor cells (MDSCs). Pancreatic tumor immune microenvironment (TIME) is typically cold, characterized by low immune infiltration, especially of tumor-infiltrating lymphocytes (TILs) and natural killers (NK). Indeed, tumor cells promote stepwise preferential recruitment of MDSCs and tumor-associated macrophages (TAMs) with an anti-inflammatory phenotype in the tumor core to achieve immune privileged and unrestricted proliferation (13). KRAS oncogenic activation, very common in PDAC, is pivotal in establishing a highly suppressive TIME by triggering the release of cytokine and chemokines which in turn promote the recruitment and polarization of myeloid and lymphoid cells with immune regulatory properties (16–18). These myeloid immune regulatory cells sculpt a T lymphocyte-hostile TME by depleting essential amino acids (Arginine, Tryptophan, Cysteine) through the coordinated action of arginase (18–20), iNOS (18), IDO1 (21, 22) by generating toxic metabolites (reactive oxygen-ROS and nitrogen species-RNS), secreting immune suppressive cytokines (IL10, TGFβ) and by ligand-receptor interactions (e.g. PDL1-PD1, FASL-FAS) (23). Conversely, increased infiltration of T lymphocytes, as well as the presence of B lymphocytes functionally organized in specific tridimensional structures, named tertiary lymphoid structures (TLSs) are associated with improved overall survival in PDAC (24, 25).

Although poor surgical chances represent a further hurdle to comprehensively solve PDAC complexity, preclinical and clinical studies demonstrated TME’s ability to support cancer progression and the prominence of combining tumor cell targeting with TME manipulation (acting on both immunologic and fibroblast compartments) to promote its conversion from a tumor-prone to an anti-tumor milieu. However, our limited understanding of the multiple heterotypic interactions among the three main compartments, coupled with the challenged of deciphering the complex heterogeneity of “subTME” composition represents the main hurdle hindering the development of novel therapeutic approaches. In this review, we envision how last-generation multi-omic technologies provided critical insights about the contribution of both CAFs and immune cells in modulating PDAC biology and current tools that can be employed to model this heterogeneity and to predict the efficacy of new therapeutic strategies.

## Genomic and transcriptomic PDAC features associated with histological and therapeutic hurdles

PDAC represents the most common types of pancreatic cancer (90% of cases) followed by neuroendocrine tumors, acinar carcinoma and pancreatoblastoma (26). PDAC derives from 2 different evolutive trajectories driven by different genetic pathways: intraductal papillary mucinous neoplasms (IPMN) are macroscopic lesions derived from the main duct or its branches, whereas pancreatic intraepithelial neoplasia (PanIN) are the most frequent early microscopic tumor lesions. Low-grade PanINs develop following proto-oncogene *KRAS* mutation and activation in ductal epithelial cells or in acinar cells triggering a de-differentiation process called acinar to ductal metaplasia (27). *KRAS* as well as G-protein alpha subunit Gas (*GNAS*) mutation and activation, together with loss of tumor-suppressor gene RING-type E3 ubiquitin ligase (*RNF43*) can drive IPMN (28, 29). Early lesions begin an evolutive path characterized by the stepwise acquisition of new features, including loss of oncosuppressor genes (e.g. *CDKN2A*, *TP53*, *SMAD4*), high grade atypia, PDAC and invasion abilities (30). Notably, preclinical models have been established resembling stepwise evolution of human PanIN and IPMN to PDAC at both genetic and histopathological levels (31, 32) allowing to study of early lesions, which are rarely identified in the clinic. *KRAS* mutation and constitutive activation are observed in the majority of PDAC. Its relevance in triggering and shaping neoplastic evolution has been confirmed in preclinical models of mutant KRAS reversible expression in which switching off the gene induces regression of both primary (33) and metastatic lesions (34) as well as promotes ductal to acinar re-differentiation (35). Accordingly, novel therapeutic strategies based on targeting mutant KRAS, including inhibitors of KRAS^G12C^ (36) KRAS^G12D^ (37), KRAS^G12D^ degraders (NCT05382559), as well as a transgenic TCR recognizing KRAS neoantigen (38) have been recently developed and currently tested in both mouse and human studies with encouraging results. Nonetheless, it has been demonstrated that neoplastic cells may acquire a KRAS independent proliferative instructions through the activation of other proto-oncogenes (39).

The biological and functional heterogeneity of PDAC can be orchestrated by tumor-cell intrinsic factors, revealing a cancer cell-driven immunosuppression. The production of the chemokine CXCL1 of cancer cells arrests T cell infiltration, thus generating a non-T- cell-inflamed TME that affects immunotherapy sensitivity (40). In human PDAC, the presence of ephrin-A receptor 2 (EPHA2) led to the upregulation of prostaglandin endoperoxide synthase 2 (PTGS2) in cancer cells, with a subsequent T cell exclusion from PDAC TME (41). The inhibition of EPHA2/PTGS2 signaling rescues T cell infiltration and may increase tumor responsiveness to immunotherapy (41). PDAC cells can shape the TIME also at epigenetic levels (42). Lysine demethylase 3A (KDM3A) regulates anti-tumor immunity through epidermal growth factor receptor (EGFR) expression in cancer cells. By acting on Krueppel-like factor 5 (KLF5) and SMAD family member 4 (SMAD4), KDM3A leads to EGFR upregulation followed by the deficiency of intratumoral T cells (42). Acquired resistance to immunotherapy response has been associated with epithelial-to-mesenchymal transition (EMT). The silencing of interferon regulatory factor 6 (Irf6) in cancer cells by EMT-transcription factors (EMT-TFs) ZEB1 and SNAIL minimize the pro-apoptotic effects of tumor necrosis factor (TNF) α (43). Hence, PDAC undertakes several mechanisms to remain refractory to immunotherapy.

Whole transcriptomic studies have been employed to leverage the characterization of both neoplastic cell and tumor contexture in PDAC. Although the limitation of achieving a bulk signature that averages the contribution of all cells in the analysis, this approach allowed to improve the characterization of tumors with apparently similar histological features. By integrating different clinical specimens, molecular technologies, and bioinformatics pipelines, a less differentiated molecular subtype of PDAC (named basal-like or quasi-mesenchymal or squamous) was identified (44, 45). The squamous subtype was associated with worse clinical outcomes (44–46), whereas the more differentiated classical subtype can be eventually subcategorized in aberrantly differentiated endocrine exocrine (ADEX), pancreatic progenitor, and immunogenic (associated with increased leucocyte infiltration) type of tumors (45). Then, laser microdissection was employed to distinguish the molecular profiles of neoplastic and stromal compartments with the final aim of establishing a computational model that may improve the definition of classical to basal subtypes and their contribution to clinical outcome in whole transcriptomic datasets (47). There is increasing evidence of TAMs role in PDAC recurrence occur often in defined cellular signaling pathways and participate in sculpting molecular subtype and increasing tumor heterogeneity (48, 49). Furthermore, integrating molecular signatures with morphological PDAC features already improved taxonomy of PDAC generating a solid soil for designing more effective therapeutic strategies (50). Nevertheless, molecular fingerprinting identification of PDAC subtypes, has not been yet employed in the clinic, for therapeutic purposes. The main reasons relate to TME plasticity that can evolve according to the stage of progression and therapeutic strategy. Moreover, several subTMEs can reside in the same tumor, and whole transcriptomics cannot take into account either TME heterogeneity or TME-neoplastic interactions. To this aim, recently, single-cell RNAseq (scRNA-seq) and first-generation spatial transcriptomic technologies were introduced. These tools allowed to increase further the resolution of both stroma and tumor cells characterization, by unveiling the spatial organization of TME as well as the cellular function and beginning the definition of heterotypic interactions between neoplastic and stromal cells (51, 52), which will be discussed in the next sections.

## Deciphering TIME in PDAC

The advent of omics, such as scRNA-seq or high-dimensional spatial analysis, has provided an unprecedented depth of knowledge on the TME complexity and heterogeneity in PDAC. A recent phenotypic and spatial immune atlas of human PDAC identifying leukocyte composition within histopathologically defined regions of surgical resections from PDAC patients allowed a new classification of PDAC based on the precise quantification of leukocyte profiles into hypoinflammed, myeloid enriched and lymphoid enriched (8). Indeed, parallel use of CyTOF, single-cell RNA sequencing, and multiplex IHC techniques has demonstrated a complex network of interaction between neoplastic and normal cell types, highlighting an inverse correlation between myeloid populations and effector CD8^+^ T lymphocytes (CTLs) (53). Multiple immune cell subsets have been shown to impact tumor biology (54). Innate immune cells represent the largest leukocyte subset detected in PDAC tumors. Myeloid cells include mostly TAMs, MDSCs and neutrophils. Importantly, the phenotype of tumor-infiltrating myeloid cells is a determinant of treatment outcome (55). TAMs arise from both infiltrating monocytes and tissue-resident macrophages (56). A limited number of studies suggest that depending on their ontogeny, TAMs have both overlapping and distinct functions in shaping the TME. Whereas monocyte-derived TAMs are more potent at sampling tumor antigens, embryonically-derived TAMs exert unique fibrosis-modulating functions which led to the production and remodeling of the extracellular matrix (ECM) (56). Indeed, TAMs show high plasticity and may be engaged in either tumor-promoting or tumor-suppressive fashion. Whereas *in vitro* TAMs have been classified into two opposite polarization states, M1-like macrophages with anti-tumor activity and M2-like macrophages with protumor properties, their phenotype *in vivo* reflects the complexity of polarization signals present within the TME (57). The presence of prostaglandin E2 (PGE2) and tumor necrosis factor (TNF) within the TME led to the conversion of tumor-infiltrating monocytes in interleukin-1β (IL-1β)-expressing TAMs (58). This population is transcriptionally enriched in inflammatory response, leukocyte recruitment and angiogenesis genes. The spatial proximity of IL-1β^+^ TAMs and cancer cells drive the acquisition of inflammatory and pathogenic properties of a subset of PDAC cells expressing an IL-1β response signature during the early stages of tumor development. The persistent inflammatory signaling in epithelial cells accelerated tumorigenesis and is associated with poor outcomes for PDAC patients (58). Hence, myeloid cells have a key role in the establishment of an immunosuppressive TME and disease progression. In a genetically engineered mouse model of PDAC, the inhibition of CSF1R^+^ TAMs resulted in a marked increase of CD8^+^ effector cells together with the reduction of collagen and hypoxia, offering a way to specifically target macrophages in PDAC (59). Toll-like receptor 9 (TLR-9) based immunotherapy was able to locally activate the immune system, converting the immune hostile into an immune permissive TME sensitizing PDAC to immunotherapy (60). In preclinical models, the CXCR1/2 inhibition was able to arrest the recruitment of macrophages and their polarization toward a tumor-promoting phenotype and increase the efficacy of anti-PD-1 (61).

Accordingly, several studies highlight the potential therapeutic benefit of redirecting myeloid cells towards antitumor and antistromal properties (55, 62). There is increasing evidence of TAMs role in PDAC recurrence. The different recurrence patterns are driven by spatially restricted tumor- and stroma-associated immune drivers, resulting in different immune cell populations and integrin networks. The spatial analysis of PDAC patients with liver recurrences emphasized an innate immune response characterized by the presence of immunosuppressive CD68^+^ TAMs localized close to tumor cells and a reduced number of CD8^+^ T cells (63). By contrast, PDAC with lung and local recurrences displayed a mix of adaptive and innate immune response. When compared to PDAC with liver recurrences, lung relapses shown an upregulation of integrin ITGAM (CD11b), which is known to inhibit immune suppression and promote antitumor immune response. Therefore, CD11b targeting could be beneficial in patients with recurrent PDACs (63).

The second major immunosuppressive cell type in PDAC is represented by MDSCs, a heterogeneous cell population composed of mature and immature cells of myeloid origin characterized by immune regulatory properties (64). During tumorigenesis, MDSCs disrupt tumor immunosurveillance by suppressing CD8^+^ T cell-mediated antitumor immunity (65–67). MDSCs can be divided into two main subgroups according to the expression of selective surface markers, the monocytic lineage (M-MDSCs) and the polymorphonuclear lineage (PMN-MDSCs); however, in humans exists an “early immature” MDSCs (eMDSC) subset (64, 68, 69). Cell plasticity and longer half-life are typical features of the monocytic-MDSC subset (70), since this population is able to differentiate into TAMs (71, 72). Tumor-released soluble factors induce an imbalanced myelopoiesis that ultimately supports MDSC generation. Several proinflammatory signals (e.g. TNFα) activates alternative molecular pathways that differentiate normal monocytes/neutrophils in MDSCs. For instance, the antiapoptotic molecule cellular FLICE (FADD-like IL-1-converting enzyme)-inhibitory protein(c-FLIP) is a main driver for M-MDSCs expansion (73, 74). Indeed c-FLIP can activate the transcription of several immunosuppression and inflammation-associated genes (i.e., Il10, Il6, Cd274, and Cd273), by alternative activation of nuclear factor kappa-light-chain-enhancer of activated B cells (NF-KB) and STAT3 activation (75, 76). Interestingly, the frequency of c-FLIP-expressing, PDL-1^+^ monocytes isolated from PDAC patients in combination with high levels of serum IL-6, has been identified as a negative independent prognostic factor for both overall survival and disease-free survival (DFS) (75). Tumor cells orchestrate MDSCs recruitment via multiple tumor-secreted factors. For instance, the intratumor levels of Regnase-1, an RNA-binding protein with endoribonuclease activity, have been negatively associated with tumor-infiltrating myeloid cells and clinical outcome in PDAC patients (77). Interestingly, Regnase-1 deletion regulates a variety of cytokines and chemokines (i.e., CXCL1, CXCL2, CSF2, and TGFβ) involved in the recruitment and education of MDSCs thus promoting PDAC progression (77). Moreover, MDSCs recruitment in the pancreatic tumor site occurs via tumor-secreted factors (e.g. CXCR2 ligands, GM-CSF and CXCL5) thus blocking the recruitment and priming of T cells (78, 79). By employing mass cytometry, crosstalk between cancer cells and PMN-MDSCs was uncovered in human PDAC (80). In myeloid-enriched and T cell-excluded contexts, PMN-MDSCs amplify inflammation and promote immune tolerance by TNF production. The PMN-MDSCs-derived TNF rewire CXCL1 overproduction by cancer cells that in turns led to dysfunction and spatial exclusion of T cells from tumor core (80). Besides soluble mediators, myeloid recruitment can occur following alterations in the expression of cell-surface molecules, such as integrins. Both MDSCs and TAMs share the expression of CD11b/CD18 integrin heterodimer (Mac-1), a key player that regulates the adhesion and migration of myeloid cells in inflamed tissues. Leukadherin-1, a small molecule activator of CD11b, was found to reduce infiltration of CD11b^+^ myeloid cells and repolarization of TAMs with a concomitant increase of CD8^+^ T cells and activated DCs in genetically engineered KPC mouse model (81). Interestingly, the activation of the antitumor response by CD11b agonist sensitizes normally resistant tumors to immunotherapies (82).

Dendritic cells (DCs) are critical for antigen cross-presentation and tumor-specific T-cell immunity. Emerging evidence demonstrates that PDAC itself can promote an immunosuppressive TME. In an engineered model neoantigen of PDAC, the disruption of immune surveillance by type I conventional dendritic cells (cDC1) led to the arrest of CD8^+^ T cells and TH_1_ activity, accelerating the neoplastic progression (15). cDC1 dysfunction begins in the early stages of PDAC, where elevated serum IL-6 affects cDC1 quantitatively and functionally, resulting in a DC semi-maturation state and a defective T cell priming (83). The use of CD40 agonist and Fms-related tyrosine kinase 3 ligand (Flt3L) enabled tumor growth control by increasing the number and activity of cDCs (83). However, more studies on the DC subset in PDAC are needed to open new therapeutic options potentially. Among the main components of the adaptive immune system, regulatory T (Treg) cells are the most abundant CD4^+^ T cell population. In humans, Tregs infiltrate starting from preneoplastic lesions to established cancer and their high prevalence has been associated with poor prognosis in PDAC (84). Despite being known as an immune suppressive population, the role of Tregs is debatable in PDAC. Various mechanisms have been proposed to define Treg as a tumor-supportive subset that led to CD8^+^ T cell suppression. For instance, tumor-infiltrating Tregs can promote immune tolerance by restraining the immunogenic functions of tumor-associated dendritic cells (DCs) necessary for CD8^+^ activation (85). In contrast, Treg depletion in a genetically engineered mouse model of PDAC accelerates tumor progression due to compensatory myeloid infiltration (86). Tregs are a key source of TGFβ ligands and their depletion results in the differentiation of inflammatory fibroblast subsets (e.g. myCAF) that increase the secretion of chemoattractants for suppressive myeloid cells (86). Other T cell populations within the TME play pro-tumorigenic roles, including Th17, Th22, CD4 and γδ T cells (87–89). During PanIN formation, CD4^+^ T cells are recruited within the tumor to arrest CD8^+^ effector functions thus contributing to tumorigenesis (90). Despite PDAC being poorly infiltrated by CD8^+^ T cells, scRNA-seq technology enabled a better understanding of this subset. A minority of CD8^+^ T cells show an exhausted transcriptional profile which escalates in advanced stages of the disease (53). Interestingly, tumors presenting both a high number of neoantigens and a strong CTL infiltrate are associated with long-term survivors, highlighting the presence of functional T cells controlling disease progression (91). Recently, the expression of immune checkpoint TIGIT was associated with a combination of immune populations (exhausted CD8^+^ T cell, Treg and NK). The CD155/TIGIT axis is essential to support immune evasion; when combined with PD-1 blockade plus CD40 activation, the CD155/TIGIT targeting stimulates a robust anti-tumor response in preclinical models of PDAC (92). Albeit less studied than T cells, B cells infiltrate and accumulate during PanIN and PDAC lesions both in mice and humans. B cells can act as either anti-tumorigenic or pro-tumorigenic populations depending on their localization and functional organization (93). Tumor-infiltrating B cells reside in TLSs, functional immune-responsive niches that have recently gained strong attention in PDAC (25, 94). When present within tumors, TLS usually associated with a most beneficial outcome (95). However, B cell fate depends on TLS maturity (96). In mature TLS, anti-tumorigenic B cells contribute to cancer immunosurveillance by producing anti-tumor antibodies and presenting tumor antigens to T cells (97). By contrast, immature TLS can originate regulatory B cells (Bregs), a pro-tumorigenic subset dispersed inside the TME and characterized by the secretion of anti-inflammatory cytokines (e.g., IL-10 and IL-35) which promote tumor progression (98, 99). In PDAC, Breg cells can restrict the activity of effector T cells while boosting Tregs, MDSCs and TAMs. For instance, Breg - FcRγ^+^ TAMs crosstalk drives a tumor-promoting macrophage phenotype supporting tumor growth (100). The spatial organization of immune cells within the TME influences survival and response to therapy in several tumor types, including PDAC (101, 102). By comparing transcriptional data of PDAC tissue samples from resected long-term and short-term survivors, a higher infiltration of B cells was observed in long-term survivors. Spatial data of long-term survivors revealed the proximity of CD20^+^ B cells and T cells with an activated effector phenotype, highlighting the importance of studying the differences in immune infiltration in a specific location (101).

**Figure 1:**
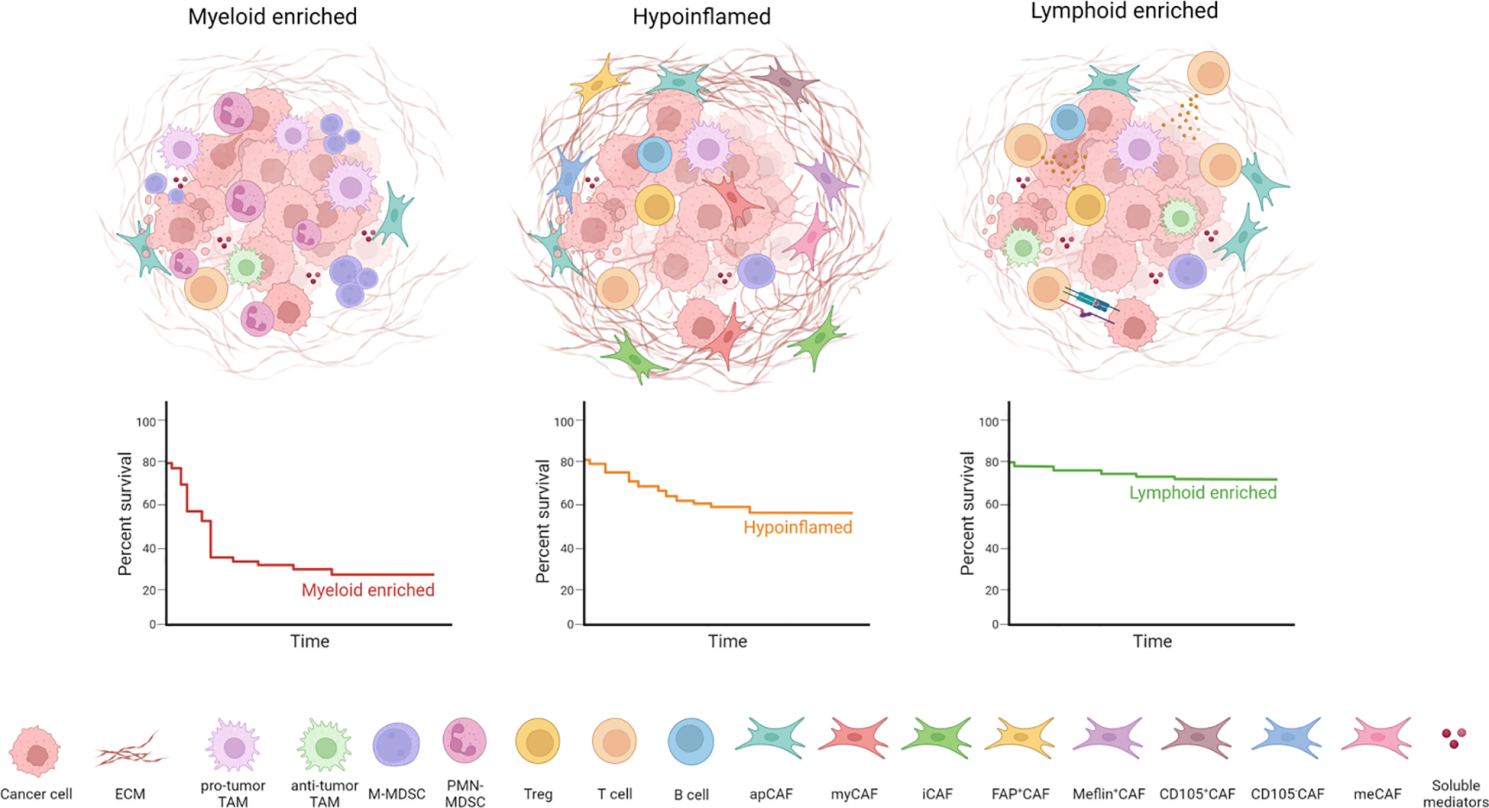
PDAC TME heterogeneity and organization. PDAC can be divided into different subtypes according to TME composition: tumor-associated macrophages (TAMs) and myeloid-derived suppressor cells (MDSCs) characterize the myeloid-enriched subtype; several cancer-associated fibroblasts (CAFs) and extracellular matrix (ECM) components determine the desmoplastic stroma typical of hypoinflammed subtype; infiltration of T cells is associated with the lymphoid-enriched subtype. The lymphoid enriched subtype holds a significant survival advantage compared to the myeloid-enriched and hypoinflamed subtype.

In summary, many differences in the immune landscape and other stromal components co-exist within individual tumors and the understanding of the spatial and dynamic relationships among diverse cell types is necessary to design accurate treatment interventions (**Figure 1**).

## Deciphering CAF heterogeneity in PDAC

One of the hallmarks of PDAC is an intense desmoplastic reaction with abundant ECM deposition which is mainly contributed by CAFs (103–105). CAFs are often the most abundant cell type in PDAC and represent a very heterogeneous population of diverse phenotypes and functions that concur to define a highly immunosuppressive TME (106, 107). The heterogeneity of CAFs emerges as the consequence of biochemical and physical signals that define sub-microenvironments with distinctive immune features in PDAC (9).

The origin of CAFs is still not fully understood, but several cellular sources have been proposed. Pancreatic stellate cells (PSCs) are specialized cells that play a crucial role in maintaining pancreas homeostasis. In their quiescent state, PSCs have a star-shaped morphology containing vitamin A lipid droplets. In response to various signals (i.e., injury or inflammation), PSCs can undergo activation, thereby losing vitamin A droplets and adopting a myofibroblast-like phenotype (108). PSCs have been considered for long the only source of CAFs, but recent studies have identified other potential sources of CAFs, including Fabp4^+^ fibroblasts, Gli1^+^ fibroblasts and Wt1^+^ fibroblasts (109–111).

Several attempts have been made to target PDAC stroma, which has been originally considered a physical barrier to drug delivery. Inhibition of the Hedgehog (HH) signaling pathway with the HH pathway inhibitor IPI-926, along with gemcitabine treatment, improved overall survival in mouse models of PDAC by increasing tumor vasculature (112). However, clinical trials using this same pharmacological scheme (gemcitabine + HH pathway inhibitor, Vismodegib) in patients with metastatic pancreatic cancer did not improve overall survival (113, 114). Subsequent preclinical studies established that genetic or pharmacological inhibition of the HH pathway promoted the emergence of poorly differentiated neoplastic phenotypes (115–117). Alternatively, the enzymatic ablation of hyaluronic acid (HA), a major component of PDAC desmoplastic stroma, restored interstitial fluid pressure in autochthonous PDAC mouse models, allowing a more efficient drug delivery associated with increased survival (118). Moreover, the combination of PEGPH20 (a clinically formulated PEGylated human recombinant PH20 hyaluronidase) and gemcitabine resulted in the inhibition of PDAC tumor growth and higher survival compared to gemcitabine treatment alone (119). Despite encouraging preclinical results, this approach was ultimately unsuccessful in clinical practice (120). The exposure of PSCs and PDAC cell lines to all-trans retinoic acid (ATRA) or vitamin D analog (calcipotriol) led to a quiescent cellular identity, *in vivo* reduced tumor growth and increased cell death (121, 122). However, calcipotriol also hampers T-cell effector functions promoting the upregulation of PD-L1, potentially compromising T-cell mediated anti-tumor response (123). To date, the effect of calcipotriol has been investigated on autoimmune diseases and a few cancer types, including breast cancer and squamous cell carcinoma. Its effect on PDAC requires still clinical investigation. The failure in translating preclinical findings based on agnostic CAFs targeting into clinical application underlined the necessity of mapping out the different phenotypes and functions of this heterogeneous stromal population. Indeed, subsequent studies have identified several CAF subtypes, with both pro-tumorigenic and anti-tumorigenic features, highlighting how stromal heterogeneity in PDAC can hinder therapeutic approaches.

Based on the antagonistic activity of TGFβ and IL1, two main phenotypes of CAF exist. myCAFs are located proximal to cancer cells where the local gradient of TGF-β reduces expression of IL1R, while iCAFs are located far from cancer cells where they sense and respond to IL1R/JAK/STAT signaling (124). If iCAFs are invariably considered pro-tumorigenic (124), myCAFs have been described with both tumor-promoting (125–127) and restraining functions (115, 117, 124). Noteworthy, these CAF subpopulations are interconvertible and able to change their phenotype according to different extracellular stimuli (128). For these reasons, the possibility of converting pro-tumorigenic CAFs into anti-tumorigenic CAFs might represent a promising therapeutic strategy. Recently, a third CAF population has been identified and named apCAFs, showing several features of the antigen presentation machinery, such as the ability to present antigens to CD4^+^ T cells, probably modulating the immune response in PDAC (129). However, since apCAFs do not express costimulatory molecules (e.g., CD80, CD86 or CD40), they cannot properly function as antigen-presenting cells. Moreover, a recent study has reported a mesothelial origin for this CAF subtype, with a potential role in immunosuppression (111). Accordingly, apCAFs induce CD8 T cell death by FASL-FAS and PDL2-PD1 interaction in breast cancer (130) and promote CD4 naïve T lymphocyte differentiation towards T regulatory cells (111). Other subpopulations of CAFs have been proposed, based on the expression of specific markers. Single-cell mass cytometric analysis has revealed that the cell surface marker CD105 can effectively distinguish two CAF populations that are functionally distinct and non-interconvertible: tumor-permissive CD105^+^ CAFs and tumor-suppressive CD105^-^ CAFs (131). The cell surface protein Meflin has been described as a marker for CAFs with tumor suppressor activity. The presence of Meflin^+^ CAFs is associated with a favorable prognosis in both human and KPC mice, whereas genetic ablation of Meflin^+^ CAFs leads to the development of poorly differentiated tumors *in vivo* (132, 133). FAP^+^CAFs can inhibit CD8^+^ T cells infiltration by expressing high levels of CXCL12 (134). The depletion of this CAFs subpopulation sensitizes these tumors to immune checkpoint therapy (106). Through single-cell RNA sequencing, an additional CAFs population expressing leucine-rich repeat containing 15 (LRRC15) and induced by TGF-β has been identified. This myCAFs subpopulation is associated with poor response to anti-PD-L1 therapy in PDAC patients (135). *In vivo* depletion of this population restrains tumor growth, reducing total fibroblast content and enhancing intratumoral CTL infiltration (136). In addition, CAFs exhibiting a highly activated metabolic state (meCAFs) have been recently identified and associated with poor prognosis and conversely to a better response to PD-1 blockade treatment in PDAC patients (137). Although CAF heterogeneity has been deeply investigated, it is still difficult to compare CAF phenotypes across species (e.g., mouse and human). Human studies investigating the presence and function of apCAFs are not available yet. Extending the definition of human CAF subsets and their functional roles is mandatory to guide the development of new therapeutic strategies.

Overall, deeper decoding of functions and features of CAFs with new technologies at single-cell resolution may foster new opportunities for stroma-targeting drugs.

## Improving the current understanding of tumor-stroma interplay with spatial biology

Spatial biology is an interdisciplinary field that combines multi-omics and imaging technologies to study the biological processes and cellular interactions inside the structures of the tissue architecture. ScRNA-seq integrated with other omics data like proteomics and Assay for Transposase-Accessible Chromatin with Sequencing (ATAC-Seq), has contributed to the discovery of rare cellular populations in both development and disease. Spatial technologies add topological information to scRNA-seq thus identifying a niche with peculiar cellular composition unraveling new pathological models and novel therapeutic targets. The resolution and complexity of these technologies have become determinants for the study of the intricated TME that sets PDAC apart from numerous other solid tumors. Xue et al. revealed novel tumor-promoting functions of Schwann cells in PDAC by employing both single-cell RNA-seq and spatial transcriptomics (138). They found that Schwann cells affect both tumor and stromal compartments toward tumor outgrowth. PDAC cells located in this area displayed typical Basal-like markers and other invasiveness markers such as metalloproteases and EMT markers. Indeed, SC-CM supports CAF polarization toward iCAF by IL1 and supports tumor proliferation. Yousuf et al. provided a holistic view of immune dysfunction in PDAC (139). They found increased amounts of inhibitory and exhausted T cells in the tumor, with consistent downregulation of cytotoxicity-related genes in CTLs placed in the tumor core compared to the ones placed at the tumor border. Infiltrating NKT cells showed an extraordinarily exhausted phenotype too, identified by the high expression of immune checkpoint proteins TIM3, LAG3, and CTLA4. Performing cell-to-cell interaction and ligand-receptor analyses on spatial transcriptomics data they identified TIGIT expression on T and NK cells correlating with the expression of its ligands PVR and PVRL2 on myeloid and tumor cells. Spatial technologies also provided new insight into the tissue remodeling and cellular population dynamics that happen during the therapy in PDAC patients. Hwang et al. integrated single nuclei RNA-seq and digital spatial profiling to characterize the cellular subtypes and spatial communities in naïve and neoadjuvant-treated PDAC patients (52). They validated the existence of previously identified clusters (44–46) exhibiting a consistent molecular pattern characterized by both classical and squamous features. Additionally, they discovered a novel cluster prevalent among patients who underwent chemotherapy, linked to poorer clinical outcomes, and labeled as “neural-like progenitor” (NRP). This study sheds light on the inter- and intratumoral diversity of pancreatic cancer, identifying treatment-associated remodeling and clinically relevant prognostication. The refined molecular and cellular taxonomy of treated PDAC samples identified in this study shed more light on the complex dynamics that happen in response to treatments highlighting novel pathways and cellular populations that may be targeted to improve standard treatment efficacy. Another study attempted to decipher insights driving chemoresistance in PDAC, integrating bulk-sequencing technologies, proteomics/phosphoproteomics, single-cell sequencing with spatial transcriptomics and high-resolution cellular imaging (51). The authors identified an iCAF increase in chemotherapy-treated patients compared to untreated ones. Moreover, they showed that specific pathways implicated in therapy resistance were upregulated in iCAFs according to the treatment. While Heat shock and AP-1 genes were more highly expressed in FOLFIRINOX samples; metallothioneins were more highly expressed in Gemcitabine + nab-paclitaxel samples. These genes are implicated in chemoresistance, suggesting a strategy to deplete iCAF via IL1β-R or JAK-STAT pathway inhibition to improve therapeutic efficacy. Besides spatial transcriptomics, other omics technologies can supply spatial definition and cues of heterotypic interactions. Proteomics has the advantage of more effectively associating data with cell phenotype. Le large et al. employed a combination of microdissection and Nanoscale liquid chromatography coupled with tandem mass spectrometry (nano LC-MS/MS) to perform spatial proteomics of both tumor and stroma regions in PDAC patients (140). They found several proteins associated with prognosis. Tumor cells from patients with poor outcomes were enriched in proteins involved in DNA replication and included in signatures of proliferation and contractility. Stroma from patients with worse survival was rich instead of proteins involved in focal adhesion processes. Moreover, they identified EPHA2 as a possible novel therapeutic target in PDAC since is highly expressed in PDAC and inhibitors are already available. Brouwer et al. employed single-cell mass cytometry and multiplex spatial IF to compare the local and systemic immunophenotype integrating samples from primary tumors, and peripheral and portal vein blood (141). They confirmed preferential infiltration of B lymphocytes and T regs in primary tumors that were confirmed as well in portal vein blood but not in circulation, and paucity of CTLs. Leveraging both mass cytometry and multiplex spatial IF they identified only in primary tumors a substantial fraction of innate lymphoid 1 like cells producing high levels of IFNγ and characterized as CD127^–^CD103^+^CD39^+^CD45RO^+^ cells.

These studies have paved the way for more detailed multi-omics profiling of the complex interactions occurring in pancreatic TME. Moreover, they identified novel pancreatic and stromal subsets enriched in patients non responding to chemotherapy, supplying a framework to better stratify patients and to identify new therapeutic targets. Since we are still in the early phase of the spatial technology era, it is easy to envision that these technologies may lead to innovative discoveries in the years to come, hopefully resulting in significant clinical improvements for pancreatic cancer patients. Nevertheless, these technologies still have significant limitations. Indeed, further development is needed both technically and, especially, computationally to improve the integration of a larger number of samples and thus achieve the identification of reliable clinical markers.

## Technology for predicting response to therapy: organoid models

Advancements in TME deconvolution at single-cell level are crucial to identifying unprecedented biochemical and functional neoplastic-stromal relationships. These technologies allow to identification of potential target candidates that can be enrolled on an *in vitro* and *in vivo* preclinical screening pipeline to be then eventually tested in clinical trials. Thus, the development of tools that can recapitulate *in vitro* (although in a simplistic way) the tridimensional organization of tumor and stromal support in providing therapeutic resistance is mandatory. Patient-derived organoids (PDO) can suit these needs. Organoids are *in vitro* self-organized 3D tissues deriving from stem cells (pluripotent, fetal, or adult). Noteworthy, the organoids can faithfully recapitulate the key functional, structural, and biological complexity of an organ *in vivo*, hence, also called “mini-organs” (142–144). To enable the *ex vivo* survival and expansion of the epithelial compartment, including stem-like cells, the organoid technology relies on two crucial components: exogenous supplementation of stromal-niche factors and extracellular matrix gels (i.e., hydrogel, Matrigel) (145). Different culture systems have been described and they can be broadly divided into Wnt -dependent (146–148) and -independent (149, 150) cultures systems (**Table 1**).

**Table 1:** Different protocols to generate and propagate pancreatic organoids.

Lab origin	Cell medium components	Extracellular matrix gel	References
Wnt-dependent			
Grapin-Botton	ROCK inhibitor, EGF, FGF1, FGF10, R-spondin 1, heparin and phorbol myristate acetate	Hydrogel	(146)
Clevers	B27, N-Acetylcysteine, gastrin, EGF, R-spondin 1, Noggin, FGF10 and Nicotinamide	Matrigel	(147)
Clevers/Tuveson	B27, N-Acetylcysteine, gastrin, EGF, R-spondin 1, Noggin, FGF10, A83-01 and Nicotinamide	Matrigel	(148)
Wnt independent			
Muthuswamy	Pancreatic Progenitor and Tumor Organoid Media (PTOM) containing DMEM with 1% B27, ascorbic acid, insulin, hydrocortisone, FGF2, all-trans-retinoic acid and Y267632	Matrigel	(149)
Skala	RPMI media supplemented with 10% fetal bovine serum, 1% penicillin-streptomycin, and EGFR	Matrigel	(150)

The culture conditions do not support the long-term propagation of native components of the tumor microenvironment. The air-liquid interface system favors the retention of native components but only to a certain extent (151). Hence, the majority of organoid culture systems lack stromal and immunological components that are key determinants of tumor biology. PDAC organotypic cultures lack TME components (e.g., fibroblasts, immune cells, endothelial cells) and several efforts have been made to better shape recapitulate the complexity of the disease by establishing co-cultures of organoids with other cell types (152, 153). Moreover, the selection of the proper culture condition is necessary to control the expression of the more appropriate transcriptional profile. This calls for important efforts in the field to further improve the culture systems by leveraging our increased understanding of physical and chemical interactions occurring with the tumor microenvironment. In pancreatic cancer as well as in other tumor types, organoid-based coculture systems have been used to recreate *ex vivo* relevant bi-directional interactions. These systems can be used to perform compartmentalized studies where individual subsets of interactions are interrogated to advance our understanding of cancer biology. It is however unlikely that organoid-based system will be able to recapitulate the cellular and physiological complexity of the native tissue. Nonetheless, it is now well established that PDOs retain the main genomic features of the parental tissue (148, 154, 155) and are exquisitely suited to understand how the microenvironment affects neoplastic cell phenotypes (156, 157). Even if the PDOs are originally established from tissue specimens which are representative of given *in vivo* subTME, exposure of PDOs to relevant TME cues might be able, at least in principle, to approximate *in vivo* cell states. That is essential to proper modulate pharmacological responses which are known to be affected by microenvironmental components. As proof of that, transcriptional cell states which are enriched in tissues from post-treatment tumors can be found *ex vivo* when PDOs are subjected to the same treatments. Hence, improving the quality of patients’ lives by acting on micro- and macroenvironmental factors holds promise in PDAC treatment (52, 158). These findings emphasize the potential of PDOs as an effective platform for drug screening in PDAC. Another interesting aspect of the organoid technology applied to pancreatic cancer is the possibility of establishing models from different disease stages (e.g., PanINs, overt carcinomas, metastatic diseases) as well as normal ductal cells, which will give the possibility of testing pharmacological sensitivities/toxicity in different settings (148, 159).

However, even if PDOs are developed from epithelial tumor cells isolated from surgical resection or tumor biopsies, which are not representative of the whole tumor, PDOs are highly dynamic cultures and are influenced by changes in TME components cell medium compositions and drug treatment (157). To obtain a PDO-derived preclinical tool for pharmacological testing and drug treatments, overcoming the limitation given by the poor representation of TME components, several approaches have been explored, including co-culture systems and on a chip-platforms (160–163).

## Current treatments and future perspectives of targeted therapies

Considering the precision oncology revolution we are experiencing, the current standard of care for PDAC has been only marginally involved. Only small subsets of patients harbor unique, actionable genetic alterations – such as homologous recombination deficiency (BRCA1/2, PALB2), mismatch repair deficiency, rare fusions (NRG1, NTRK) – granting them access to potentially beneficial targeted therapies (164). For the others, the mainstay of treatment relies on multiagent combination chemotherapeutic regimens (FOLFIRINOX and Gemcitabine/nab-paclitaxel), according to disease stage and patient fitness (165).

**Figure 2:**
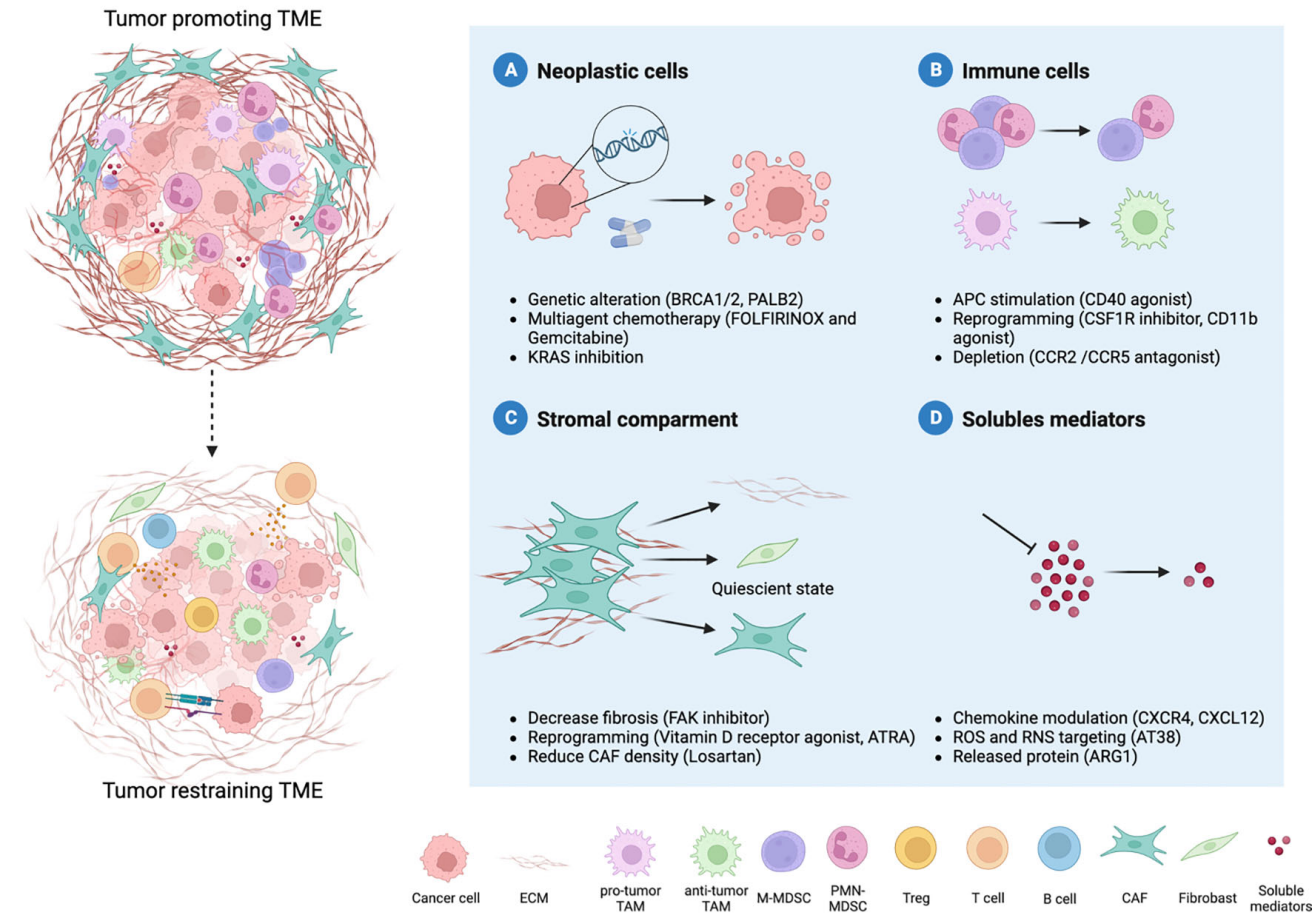
Therapy-induced reprogramming of PDAC TME. The combination of different therapeutic strategies can revert the hostile microenvironment towards an anti-tumor one. Four major therapeutic interventions are emerging to improve PDAC treatment: neoplastic cells, immune cells, stromal compartments and soluble mediators. Several approaches are focused on neoplastic cells and their genetic alterations **(A)**. Moreover, the microenvironment that surrounds PDAC cells holds key insight into novel treatment options. The reprogramming of myeloid cells can relieve immune suppression **(B)** and coupled with increased antigen presentation allow the recruitment and priming of T cells. Another strategy is represented by the modulation of the desmoplastic stroma, such as cancer-associated fibroblasts (CAFs) **(C)**. Given the double role of CAFs in the TME, reverting the tumor-promoting into quiescent cells represent the best strategy. Indeed, multiple cell types co-exist within the TME, and their communication occurs through soluble mediators (metabolites, cytokines, chemokines) **(D)**. Targeting soluble mediators can hamper immune cells recruitment and pro-tumor differentiation, as well as deposition of extracellular matrix, thus limiting tumor progression.

However, major preclinical research efforts to decode the complex interplay between cancer cells and the TME have identified new molecular vulnerabilities, opening the door to novel targeted therapies whose efficacy is currently under investigation in several clinical trials (**Figure 2**; **Table 2**).

**Table 2:** Selected trials in PDAC by mechanisms or target.

Mechanism or target	Preclinical rationale	Agent	Combination	Patient population	NCT trials	Ph	Status
Stroma reprogramming							
**FAK inhibitor**	↓fibrosis ↓T_regs_ ↑CD8^+^T cells	Defactinib	Pembrolizumab SBRT Pembrolizumab + gemcitabine	R LA advanced	NCT03727880 NCT04331041 NCT02546531	2 2 1	Active-R Active-R Completed (166)
		GSK2256098	Trametinib (MEK inhibitor)	advanced	NCT02428270	2	Completed(167)
**IL-6 antagonist**	↑CD8^+^T cells ↓*α*SMA^+^ cells	Siltuximab	Spartalizumab	M	NCT04191421	1/2	Completed
		Tocilizumab	GnP Nivolumab + Ipilimumab + XRT GnP + atezolizumab	advanced advanced M	NCT02767557 NCT04258150 NCT03193190	2 2 1/2	Completed Completed (168) Completed (169)
**Vitamin D Receptor agonist**	Reverts CAFs into a quiescent phenotype ↓fibrosis ↑PD-L1	Paricalcitol	GnP Pembrolizumab ± GnP Nivolumab + GnP + cisplatin GnP Pembrolizumab (maintenance)	R R M M M	NCT02030860 NCT02930902 NCT02754726 NCT03520790 NCT03331562	1 1 2 2 2	Completed Completed Active-NR Active-NR Completed
**ATRA**	Reverts CAFs into a quiescent phenotype ↑CD8^+^T cells	ATRA	GnP GnP	advanced LA	NCT03307148 NCT04241276	1 2	Completed (170) Active-NYR
**VEGF inhibition**	↓angiogenesis ↓T cell exhaustion ↑DCs maturation ↑endothelial cells LAMs ↑effector leukocytes infiltration	Lenvatinib	Pembrolizumab Pembrolizumab (maintenance) Durvalumab, GnP H-101, tislelizumab	advanced advanced advanced advanced	NCT03797326 NCT04887805 NCT05327582 NCT05303090	2 2 1/2 1b	Active-NR Active-R Active-R Active-R
		Bevacizumab	GnP, atezolizumab	M	NCT03193190	1/2	Active-R
**Integrin inhibition**	Blockage association *α*_5_ *β*_1_ integrin + fibronection ↓angiogenesis	Volociximab	Gemcitabine	M	NCT00401570	2	Completed
**Integrin cytotoxin**	Apoptosis of integrin-*α*_V_ *β*_3_-expressing cells	ProAgio		advanced	NCT05085548	1	Active-R
**Hyaluronan dissolution**	↓collapse functional blood vessels ↑delivery molecules & therapeutics to cancer cells	PEGPH20	Gemcitabine GnP GnP FFX XRT + Gemcitabine Cetuximab Atezolizumab	M M M M LA R M	NCT01453153 NCT01839487 NCT02715804 NCT01959139 NCT02910882 NCT02241187 NCT03193190	1/2 2 3 1/2 2 NA 1/2	Completed (171) Completed (120, 172) Completed (173) Completed (174) Completed Completed Active-R (169)
**Angiotensin II receptor blockade**	↓fibrosis ↓collagen I and hyaluronan deposition ↓CAFs density ↓vascular collapse ↑drug delivery ↓VEGF expression	Losartan	FFX + XRT FFX + SBRT + Surgery ± Nivolumab Hypofractionated RT FFX + Elraglusib (9-ING-41; GSK-3*β* inhibitor) Paricalcitol + HCQ mFFX ± switch to GnP followed by Capecitabine/XRT	LA R/BR/LA BR/LA M R R/BR/LA	NCT01821729 NCT03563248 NCT04106856 NCT05077800 NCT05365893 NCT04539808	2 2 1 2 1 2	Completed (175) Active-NR Active-R Active-R Active-R Active-R
**CEND-1**	Tumor penetrating peptide (via *α*_V_ receptor binding) + trans tissue transport pathway activation (via neuropilin binding) ↑drug delivery	CEND-1	GnP GnP FFX ± Panitumumab	M M R/BR	NCT03517176 NCT05042128 NCT05121038	1 2 1b/2	Completed (176) Active-R Active-R
Immune cells targeting							
**CD40 agonist**	↑stimulation APCs ↑CD8^+^ T cells activation and maturation (independent from CD4^+^ T cells) ↑TAM polarization (from M2 to M1 phenotype)	Selicrelumab	Gemcitabine Perioperative XRT (Gemcitabine) GnP GnP + Atezolizumab	advanced R R advanced	NCT00711191 NCT01456585 NCT02588443 NCT03193190	1 1 1 1/2	Completed (62) Completed Completed Active-R
		Sotigalimab	GnP + Nivolumab	M	NCT03214250	1b/2	Completed (177, 178)
		CDX-1140	CDX-301 (FLT3L)	R	NCT04536077	1	Active-R
		SEA-CD40	Pembrolizumab ± GnP	advanced	NCT02376699	1	Completed (179)
		Mitazalimab	mFFX	M	NCT04888312	1/2	Active-NR
**Oncovirus**	Oncolytic adenovirus modified to include additional immune system stimulators (CD40L and 4-1BBL)	LOAd703	GnP ± Atezolizumab Chemotherapy (GnP or Gemcitabine)	advanced advanced	NCT02705196 NCT03225989	1/2 1/2	Active-R Active-NR
**CXCR4 antagonist**	↓cancer cells invasion potential ↓angiogenesis (VEGF-independent) ↓PD-1 ICIs resistance ↑ CD8^+^ T + NK cells infiltration ↓CAFs, MDSC, T_regs_	Plerixafor	–	advanced	NCT02179970	1	Completed
		Motixafortide	Cemiplimab Pembrolizumab Pembrolizumab ± nal-IRI/5FU Cemiplimab + GnP	M M M M	NCT04177810 NCT02907099 NCT02826486 NCT04543071	2 2 2 2	Completed Completed Completed (180, 181) Active-R
**CXCL12 antagonist**	↑ CD8^+^ T + NK cells infiltration ↑PD-1 ICIs activity	Olaptesed Pegol	Pembrolizumab Pembrolizumab ± NalIRI/5FU or GnP	M M (MMS)	NCT03168139 NCT04901741	1/2 2	Completed (182) Active-NYR
**CSF-1R inhibitor**	↓TAM ↑TAM polarization (from M2 to M1 phenotype) ↑ICIs activity ↑effector/regulatory T cells ratio	IMC-CS4	Pembrolizumab, GVAX, cyclophosphamide	BR	NCT03153410	1	Active-NR
		Pedixartinib	Durvalumab	advanced	NCT02777710	1	Completed
		AMG820	Pembrolizumab	advanced	NCT02713529	1/2	Completed (183)
		Cabiralizumab	Nivolumab ± chemotherapy (GnP or nal-IRI + 5FU/LV or FOLFOX)	advanced	NCT03336216	2	Completed
**CD11b agonist**	↓myeloid migration into tissue ↑TAM polarization (from M2 to M1 phenotype)	ADH-503	Pembrolizumab or GnP	advanced	NCT04060342	1/2	Completed
**STING agonist**	↑CTLs priming against cancer cells (via type I IFN DCs activation) ↓TAM, MDSC, T_regs_	TAK-500	± Pembrolizumab	advanced	NCT05070247	1	Active-R
		ulevostimag	± Pembrolizumab	advanced	NCT03010176	1	Completed
**CCR2 antagonist**	↓TAM ↑TILs	PF-04136309	mFFX GnP	BR M	NCT01413022 NCT02732938	1 1/2	Completed (184) Terminated (185)
		CCX872-B	FFX	advanced	NCT02345408	1	Completed
**CCR2/CCR5 dual antagonist**	↓TAM ↑TILs	BMS-813160	± Nivolumab ± chemotherapy (GnP or II line)	advanced	NCT03184870	1/2	Completed
**CD73/Adenosine receptor inhibition**	↑CD8^+^ T cells ↑PD-1 ICIs activity	Oleclumab	± durvalumab durvalumab ± chemotherapy (I line GnP or II line mFOLFOX)	advanced M	NCT02503774 NCT03611556	1 1/2	Completed (186) Completed
		Taminadenant	± Spartalizumab	advanced	NCT03207867	2	Terminated
		NZV930	± Spartalizumab	advanced	NCT03549000	1	Terminated
		Quemliclustat	GnP ± Zimberelimab	M	NCT04104672	1	Active-NR
Adoptive cellular therapy							
**T Cell Receptor (TCR) therapy**	Tumor antigen targeted cytotoxic T cell activity mediated by restricted TCR-engineered immune effector cells	TCR-T	Pembrolizumab + CDX-1140	advanced	NCT04520711	1	Active-NR
		Anti-KRAS G12V murine-TCR PBL	cyclophosphamide + fludarabine + high-dose aldesleukin	M	NCT03190941	1/2	Active-R
		Mutant KRAS G12V-specific TCR transduced autologous T cells	cyclophosphamide + fludarabine ± anti-PD-1	advanced	NCT04146298	1/2	Active-R
**Tumor- Infiltrating Lymphocytes (TILs)**	Unaltered TILs expansion and transfer	Autologous TILs	Pembrolizumab cyclophosphamide + fludarabine cyclophosphamide + fludarabine + IL2	M advanced/recurrent recurrent/refractory	NCT01174121 NCT03935893 NCT03610490	2 2 2	Active-R Active-R Active-NR
**CAR-T therapy**	Tumor antigen targeted cytotoxic T cells activity mediated by a Chimeric Antigen Receptor introduced into the immune effector cells	mesothelin-CAR-T cells	- cyclophosphamide -	advanced advanced R	NCT03323944 NCT03638193 NCT06054308	1 NA NA	Active-R NA NYR
		TnMUC1-CAR-T cells	cyclophosphamide + fludarabine	M	NCT04025216	1	Terminated
		CEA-CAR-T cells	- - -	advanced advanced advanced	NCT05396300 NCT05415475 NCT06010862	1 1 1	Active-R Active-R Active-R
		Claudin18.2-CAR-T cells	- - -	advanced advanced advanced	NCT05472857 NCT04404595 NCT05539430	1 1 1	Active-R Active-R Active-R
Vaccine							
**mRNA neoantigen vaccines**	↑APCs ↑neoantigen-specific-T cells	Autogene Cevumeran	Atezolizumab + mFFX Atezolizumab + mFFX	Resected Resected	NCT04161755 NCT05968326	1 2	Active-R (187) Active-R
**GM-CSF producing vaccines**	↑APCs ↑cytotoxic T cell-mediated immune response	GVAX	Nivolumab + Urelumab (anti-CD137) ICIs (Nivolumab + Ipilimumab) cyclophosphamide +Nivolumab + SBRT cyclophosphamide +Pembrolizumab + SBRT	R M BR LA	NCT02451982 NCT03190265 NCT03161379 NCT02648282	2 2 2 2	Active-R (188) Completed Active-NR Completed
**Peptide vaccines**	↑APCs ↑neoantigen-specific-T cells	Personalized	Imiquimod ± Pembrolizumab ± Sotigalimab	advanced	NCT02600949	1	Active-R
		GRT-C903/GRT-R904	ICIs (Nivolumab + Ipilimumab)	M	NCT03953235	1/2	Completed
		CV301	Durvalumab + Capecitabine	M	NCT03376659	2	Terminated

anti-PD-L1 inhibitor (Pembrolizumab; Durvalumab; Atezolizumab); anti-PD-1 inhibitor (Nivolumab, Spartalizumab; Cemiplimab; Tislelizumab; Zimberelimab); anti-CTLA4 inhibitor (Ipilimumab; Tremelimumab). ↓ decreased presence/expression, ↑ increased presence/expression.

Lessons learned from the first testing of stroma-depleting drugs have shaped the understanding of CAF biology and their double-edge role in promoting or restraining tumor progression (115). Therefore, subsequent trials have aimed at reverting tumor-supportive cells into quiescence or reprogramming into an anti-tumor phenotype. The disappointing results of single-agent immune checkpoint inhibitors (ICIs) in unselected patient populations paved the way for developing multimodal combinatorial strategies to overcome therapeutic resistance in PDAC and maximize clinical benefit (189). However, improved clinical activity compared to standard-of-care treatments has yet to be demonstrated. Current boundaries to these combinatorial approaches include limited availability of preclinical tumor models that faithfully represent the complex interaction between cancer-, immune- and stromal cells in the TME, the absence of early detection diagnostic and predictive and prognostic biomarkers to detect early disease and identify patients with the greatest chance of response and the likelihood that even minimal variation in dosing schedule, treatment duration and/or drug sequencing might alter the anti-tumor efficacy of the combination (190). Considering the improved definition of PDAC subtypes and increased availability of drugs targeting neoplastic and stromal (immune and fibroblast) cells, the “traditional” Phase 1 to 3 trial progression might not be appropriate (191). Thus, new protocol designs have emerged (192), including “basket” trials (in which patients with solid tumors of different histology but with shared features - e.g. specific biomarkers - are put together in the same study), and “umbrella” platforms (in which patients with same pathology are divided in different arms according to the presence of peculiar biomarkers). For instance, MORPHEUS-PDAC (NCT03193190), is an umbrella trial designed to evaluate ten experimental arms with various Atezolizumab combinations with two comparators arms (GnP or mFOLFOX) in the metastatic setting; whereas Precision Promise (NCT04229004), PIONEER-Panc (NCT04481204), REVOLUTION (NCT04787991) and the GVAX immunotherapy (NCT02451982) trials will test multiple investigational combinations in parallel against standard of care arms.

Continue efforts in integrated pre-clinical and clinical research and collaborations between stakeholders are essential to identify new effective treatments and to implement a biomarker-based selection of cytotoxic chemotherapy, targeted therapy, and immunotherapy to improve the survival of PDAC patients.

## Future directions

PDAC therapy is undergoing a paradigm shift by applying new small-drugs and immunological approaches. Characterize the genetic profile of cancer cells (intrinsic properties) but also the immune contexture of tumor microenvironment (extrinsic properties) is mandatory to optimize the effectiveness of these approaches, since cancer and stromal cells are continuously in communication and are capable of bidirectional influence, reshaping each other’s cell properties (58, 193, 194). It is currently unclear how PDAC immune landscape evolves as tumors acquire additional genomic alterations as well as the drivers shaping TME immune complexity in primary tumors versus distant metastases (8) but both information are essential for patient stratification. Therefore, one of the major goals in the near future is the definition of new biomarker able to take into account both intrinsic and extrinsic PDAC features. To maximize and speed up the bench to patients ‘bed translation, it is essential to integrate better preclinical and clinical research. Essential achievements in improving the knowledge of PDAC evolution has been obtained by generating PDAC-disease models (31, 195), PDAC *in vitro* screening cell systems (148, 196), new chemical drugs and inhibitors (197), TME datasets (52, 139, 198) and genomic/molecular tumor profiles (45, 91, 199), as well as advance in biotechnology and clinical research allowed to employ cutting-edge approaches, including personalized RNA neoantigen vaccines (200) and selective inhibitors in PDAC treatment (201, 202). However, results from immunotherapy clinical trials in PDAC have thus far been modest, with clinical benefit in a small subset of MMR-D PDAC. In a comprehensive report from 2022, a revision was conducted on all interventional studies for PDAC completed between 2010 and 2020, as documented in the ClinicalTrials.gov registry— the US National Library of Medicine’s database for clinical trials. The findings revealed that out of 551 trials included in the study, 165 (30%) lacked available results. Among the remaining 297 trials with accessible results published in full-text articles, the median duration between the primary completion date and the date of publication was 47.6 months, with a 95% confidence interval of 39.6 to 61.9 months (203). Collectively, these results pinpoint that clinical trial results, including clinical trials with negative outcomes, should be published in a timely manner to support inform and improve future trial design changing both parameters for patient recruitment and combination therapies.

Emerging evidence point out that the state of well-being of the patient influences treatment response in cancer patients, including PDAC (204). Lifestyle interventions, such as exercise and nutrition, can improve the quality of life of PDAC patients by meeting their physiological needs (204). For instance, aerobic exercise can amplify the immune-cell mediated cytotoxicity on pancreatic cancer cells to reduce PDAC growth and enhance sensitivity to both immunotherapy and standard-of-care chemotherapy (205). Also, dietary interventions can influence immune responses since the growth and viability of cancer cells depend on nutrient availability. A strong anti-tumoral effect was described in KPC mice undergoing ketogenetic diet (206). When combined with gemcitabine, a ketogenetic diet contributes to increment the effectiveness of chemotherapy (206). Hence, improving the quality of patients’ lives by acting on micro- and macroenvironmental factors holds promise in PDAC treatment.

Pursuing high-risk research with high-reward potential may advance the development of promising personalized combination therapies. To this aim, integrating acknowledged (e.g. chemo and radiotherapy-based) and novel neoplastic targeting approaches [including passive and active immunotherapeutic strategies (200, 207–210)] with TME targeting solutions (211) and additional research fields, which could not be addressed in this review, such as cancer metabolism (212), vessel remodeling (213, 214), CAR T cell therapies (215) have good promises as potential strategies for PDAC therapy. We aspire to transform PDAC from a silent, formidable threat into a manageable and treatable disease in the near future.

## Author contributions

CM: Writing – original draft, Writing – review & editing. FL: Writing – original draft, Writing – review & editing. AA: Writing – original draft, Writing – review & editing. GL: Writing – original draft, Writing – review & editing. MB: Writing – original draft, Writing – review & editing. SP: Writing – original draft, Writing – review & editing. CC: Writing – original draft, Writing – review & editing. VC: Writing – original draft, Writing – review & editing. SU: Writing – original draft, Writing – review & editing. FS: Writing – original draft, Writing – review & editing, Supervision.
